# Breaking the Solubility-Permeability Tradeoff: Surfactant-Mediated Enhancement of Oral Etoposide Absorption

**DOI:** 10.3390/biom16091251

**Published:** 2026-08-28

**Authors:** Noa Fine-Shamir, Avital Beig, Arik Dahan

**Affiliations:** Department of Clinical Pharmacology, School of Pharmacy, Faculty of Health Sciences, Ben-Gurion University of the Negev, Beer-Sheva 8410501, Israel; noa.f.shamir@gmail.com (N.F.-S.); london0631@gmail.com (A.B.)

**Keywords:** oral drug delivery, solubility-enabling formulation, solubility-permeability interplay, nonionic surfactants, P-gp inhibition, bioavailability enhancement, BCS

## Abstract

Developing effective oral formulations for poorly soluble anticancer drugs remains a major pharmaceutical challenge due to the combined limitations of solubility, permeability, and efflux transporter activity. In this work, we investigated the influence of the nonionic surfactants Cremophor EL, Pluronic P-85, and Pluronic F-68 on the solubility and intestinal permeability of the anticancer drug etoposide. All surfactants significantly increased etoposide aqueous solubility. While in vitro permeability across an artificial membrane demonstrated the expected solubility-permeability tradeoff, in vivo SPIP studies in rats revealed a distinctive solubility-permeability interplay for Cremophor EL and Pluronic P-85, which simultaneously enhanced solubility and permeability, likely through P-gp inhibition. In contrast, Pluronic F-68 exhibited the classical solubility-permeability tradeoff, consistent with its reported negligible P-gp inhibitory activity. Mechanistic analysis indicated that surfactant hydrophobicity and molecular weight critically influence P-gp inhibition via ATPase modulation. Surfactants with higher hydrophobicity and moderate molecular weight can integrate into the phospholipid bilayer, enabling direct interaction with P-gp and disruption of its ATPase function. These findings provide strategic insights for the rational design of oral formulations capable of overcoming the solubility-permeability tradeoff, improving the bioavailability of challenging anticancer drugs, and may facilitate the transition from intravenous to oral chemotherapy.

## 1. Introduction

The two principal determinants of oral drug absorption are the solubility/dissolution of the drug within the aqueous intestinal environment and its permeability across the intestinal epithelium [1,2,3]. Both properties may be profoundly influenced by formulation components. In particular, solubility-enabling excipients are widely employed in oral formulations of lipophilic drugs; however, despite their intended role in improving apparent solubility, it is now well recognized that these excipients may simultaneously alter drug permeability [4,5,6]. This dual effect may explain why formulations that successfully enhance the apparent solubility of poorly soluble drugs do not necessarily improve overall oral absorption [7,8,9,10,11,12,13,14]. Indeed, our previous studies demonstrated that the most common outcome of solubilization-based formulation strategies is a solubility-permeability tradeoff, characterized by an inverse relationship between apparent solubility and permeability [15]. Such behavior has been reported for formulations based on cyclodextrins [16,17,18], surfactants [19,20,21], cosolvents [22,23,24], hydrotropes [25], and copolymer-based systems [26,27]. In these systems, enhanced apparent solubility was typically accompanied by reduced apparent permeability, and the balance between these opposing effects ultimately dictated formulation success in terms of oral absorption and bioavailability.

Conversely, formulations capable of generating and maintaining supersaturation, such as amorphous solid dispersions, have been shown to exhibit a more favorable solubility-permeability interplay, whereby permeability remains largely unaffected despite substantial increases in supersaturation levels [28,29,30,31]. While these relationships are relatively well understood for passively permeating drugs, the interplay becomes considerably more complex when active transport processes contribute to intestinal drug absorption [32,33].

Drug permeability across the intestinal membrane may occur through passive diffusion or carrier-mediated transport mechanisms. Passive transport, driven by the concentration gradient across the intestinal membrane, predominantly occurs via the transcellular route, although paracellular transport may also contribute [34,35]. In contrast, active transport processes require energy consumption and may either facilitate drug uptake or reduce absorption through efflux mechanisms [36]. Since overall effective intestinal permeability represents the combined contribution of passive and active transport pathways (P_Total_ = P_Passive_ + P_Active_) [37], interactions between formulation excipients and membrane transporters can markedly alter the solubility-permeability interplay. For example, vitamin E TPGS has been shown to simultaneously enhance both the solubility and permeability of lipophilic P-glycoprotein (P-gp) substrates, owing to its combined solubilizing capacity and inhibitory effect on P-gp-mediated efflux [38,39]. Recent studies have further demonstrated that pharmaceutical excipients can modulate intestinal drug transporters, highlighting that excipients may have effects on drug disposition beyond their traditional roles as solubilizers or formulation aids [40,41,42].

The present study aimed to elucidate the factors governing the performance of solubility-enabling formulations containing three widely used pharmaceutical excipients: Cremophor EL, Pluronic P-85, and Pluronic F-68. These nonionic surfactants are extensively utilized due to their high solubilization capacity, low toxicity, and favorable biocompatibility profiles. Cremophor EL, a nonionic emulsifier and solubilizer produced by ethoxylation of castor oil, is commonly employed in formulations used in oncology, transplantation medicine, and anesthetic applications. Beyond its ability to enhance the aqueous solubility of lipophilic drugs, both in vitro and in vivo studies have demonstrated significant inhibition of P-gp-mediated efflux by Cremophor EL [43,44,45,46,47,48,49], resulting in improved oral bioavailability of drugs such as saquinavir in humans [50,51].

Pluronic block copolymers are amphiphilic triblock copolymers composed of polyethylene oxide (PEO) and polypropylene oxide (PPO) segments arranged in a triblock (EOx–POy–EOx) structure. Variations in the relative lengths of the hydrophilic EO(x) and hydrophobic PO(y) units generate polymers with diverse hydrophilic–lipophilic balance (HLB) values and physicochemical properties [52]. Owing to their amphiphilic nature, Pluronic copolymers are widely used in pharmaceutical formulations as solubilizers, emulsifiers, viscosity modifiers, and drug delivery vehicles [26,53]. Importantly, certain Pluronic polymers have been reported to inhibit P-gp activity, whereas others lack such effects [53,54,55]. Thus, despite belonging to the same excipient family, individual Pluronic polymers may exhibit distinct solubility-permeability interplay patterns [26,33,56]. These observations underscore the importance of rational, case-specific excipient selection during formulation development [56,57,58].

To complement the in vitro permeability assessment, the single-pass intestinal per-fusion (SPIP) model in male Wistar rats was used to evaluate etoposide permeability across an intact intestinal mucosa under in vivo conditions. This model preserves key physiological and transporter-mediated processes that are not fully represented by artificial membrane models and shares important features with the human intestine, including epithelial barrier function and P-gp-mediated efflux.

To investigate the factors dictating the success or failure of solubility-enabling formulations containing Cremophor EL, Pluronic P-85, or Pluronic F-68, we evaluated the concentration-dependent effects of these excipients on the solubility, in vitro permeability, and in vivo rat intestinal permeability of the antineoplastic agent etoposide. Etoposide is a poorly water-soluble anticancer drug and a known substrate of P-gp-mediated efflux, exhibiting low and highly variable oral bioavailability [39,59,60]. Consequently, etoposide is administered predominantly by the intravenous route. Identifying formulation strategies capable of improving its oral absorption may therefore support the ongoing transition from injectable to oral chemotherapy regimens. The solubility-permeability interplay associated with each excipient was systematically analyzed to advance mechanistic understanding of excipient-mediated effects on drug absorption and to support more rational formulation design for poorly soluble anticancer drugs.

## 2. Materials and Methods

### 2.1. Materials

Etoposide, MES buffer powder, Pluronic copolymers (P-85 and F-68), trifluoroacetic acid (TFA), and DMSO were purchased from Sigma Chemical Co. (St. Louis, MO, USA). Cremophor EL was purchased from BASF (Ludwigshafen, Germany). Pre-coated PAMPA Plate System was obtained from CORNING (Two Oak Park, Bedford, MA, USA). UPLC-grade acetonitrile and water were obtained from Merck KGaA (Darmstadt, Germany). All other chemicals were of analytical reagent grade.

### 2.2. Solubility Experiments

The solubility of etoposide as a function of increasing concentrations of Cremophor EL, Pluronic P-85, or Pluronic F-68 was tested using the shake-flask method, as previously described [61]. Briefly, excess amounts of etoposide were added to glass vials containing MES buffer solution (pH 6.5) with varying concentrations of each excipient (n = 4). The vials were placed in a shaking water bath (100 rpm) at room temperature (25 °C) for 48 h to reach equilibrium, centrifuged (14,000 rpm) for 15 min, and the supernatant was immediately assayed for etoposide content by UPLC.

### 2.3. PAMPA Studies

A pre-coated PAMPA (Parallel artificial membrane permeability assay) system was used to study the in vitro permeability of etoposide from solutions containing Cremophor EL, Pluronic P-85, or Pluronic F-68 solutions, using pre-coated 96-well filter plates, as previously described [62]. Briefly, donor plate wells (300 μL/well) were filled with etoposide solutions prepared in 10 mM MES buffer (pH 6.5) with increasing concentrations of each excipient (Cremophor EL, Pluronic P-85, or Pluronic F-68). Etoposide initial concentration in all donor wells was maintained at 50% of the equilibrium solubility, to ensure similar thermodynamic activity across all experimental groups. Receiver plates (200 µL/well) were filled with 10 mM MES buffer. The donor and receiver plates were coupled, and the assembled sandwich was incubated at room temperature for 2.5 h. The receiver samples were then collected and immediately assayed for etoposide content by UPLC. The coefficients of PAMPA permeability studies were calculated according to the manufacturer’s instructions, using the following Equation (1):

(1) $$P=\frac{\left\{-\ln\left[1-\frac{C_{A}\left(t\right)}{C_{eq}}\right]\right\}}{\left[A*\left(\frac{1}{V_{D}}+\frac{1}{V_{A}}\right)*t\right]}$$

where C_0_ is the initial compound concentration in the donor well (mM), A is the filter area (0.3 cm^2^), V_D_ is the donor well volume (0.3 mL), V_A_ is the acceptor well volume (0.2 mL), t is the incubation time, and C_A(t)_ is the compound concentration in the acceptor well at time t. C_eq_ = [C_D(t)_ × V_D_ + C_A(t)_ × V_A_]/(V_D_ + V_A_), where C_D(t)_ and C_A(t)_ are compound concentrations in the donor and acceptor wells at time t, respectively. Mass balance was regularly validated at the experimental endpoint by comparing the total drug amount in donor and acceptor wells to the initial dose; total recovery exceeded 98% in all experimental groups. Membrane integrity was validated by obtaining unmeasurable permeability of Lucifer yellow across all experimental conditions.

### 2.4. Single-Pass Intestinal Perfusion (SPIP) Studies in Rats

Single-pass intestinal perfusions (SPIP) permeability studies in rats were conducted to investigate the impact of increasing levels of Cremophor EL, Pluronic P-85, or Pluronic F-68 on the rat intestinal permeability of etoposide in vivo. All animal experiments were conducted in accordance with ethical guidelines and approved by the Ben-Gurion University of the Negev Animal Use and Care Committee (Protocol IL-08-01-2015). Adult male Wistar rats (Harlan, Israel) weighing 300–350 g were housed and handled according to the Ben-Gurion University of the Negev Unit for Laboratory Animal Medicine Guidelines. The animals were not genetically modified, and no previous experimental procedures were performed on the animals before the SPIP experiments. Rats were fasted overnight (12 h) prior to each experiment, with free access to water.

Single-pass intestinal perfusion (SPIP) studies in the jejunum segment, were performed according to a previously described method [63]. Briefly, rats were anesthetized (1 mL/kg ketamine/xylazine intramuscular injection) and two cannulas were placed in the two ends of the tested segment. The intestinal segment was thoroughly rinsed with normal saline for at least 10 min to remove residual intestinal contents and avoid potential interference from endogenous luminal components during the experiment. The integrity of the intestinal mucosa was preserved in all experimental groups, as was validated by negligible permeability of the non-absorbable marker (phenol red). Three rats were used for each experimental group (Pluronic F-68, Cremophor EL, and Pluronic P-85), resulting in a total of nine animals used in the study. The nine rats were allocated to the three experimental groups using a predetermined systematic allocation sequence. Rats were assigned sequentially to the three solutions in a repeating order: solution 1 (Cremophor EL) was administered to rats 1, 4, and 7; solution 2 (Pluronic P-85) was administered to rats 2, 5, and 8; and solution 3 (Pluronic F-68) was administered to rats 3, 6, and 9. The investigators were aware of the group allocation during animal allocation and throughout the conduct of the SPIP experiments. The group allocation was also known during outcome assessment and data analysis. No blinding procedure was implemented.

All animals that underwent the SPIP procedure were included in the analysis, and no animals were excluded based on predefined inclusion or exclusion criteria. All experimental groups were subjected to the same experimental and analytical procedures. Potential confounding factors related to treatment administration order, animal/cage location, and measurement order were not specifically controlled or randomized.

After the intestinal segment was thoroughly rinsed, the tested solutions were pumped at a flow rate of 0.2 mL/min through the jejunal segment, first for 30 min to ensure steady-state conditions, and then for an additional 90 min, during which the samples were collected at 10 min intervals. Etoposide initial concentration in the perfused solution was maintained at 50% of the equilibrium solubility, to ensure similar thermodynamic activity across all experimental groups. The collected samples were centrifuged and immediately tested for etoposide content by UPLC. The effective permeability (*P*_eff_) through rat jejunum in the SPIP studies was determined by the following Equation (2):

(2) $$P_{eff}=\frac{-Qln(\frac{{C^{\prime }}_{out}}{{C^{\prime }}_{in}})}{2\pi RL}$$

where Q is the perfusion flow rate (0.2 mL/min), C′_out_/C′_in_ is the ratio of etoposide outlet vs. inlet concentrations that have been corrected for water flux using the gravimetric method, R is the radius of the jejunum (0.2 cm), and L is the exact length of the rinsed segment. Mean values and standard deviations (SD) were calculated using Microsoft Excel.

### 2.5. Theoretical Permeability Predictions

We have previously shown that the predicted apparent permeability of etoposide from different levels of solubilizer is a function of a tradeoff relationship between the increased drug solubility afforded by a given excipient level and the consequent decreased permeability [64]. Accordingly, the predicted apparent permeability of etoposide from formulations containing different levels of Cremophor EL, Pluronic P-85, or Pluronic F-68 was calculated using the inverse correlation equation: Pm = Pm(0)·Saq(0)/Saq in which Pm(0) is the intrinsic membrane permeability of etoposide (either in vitro (PAMPA) or in vivo), Saq(0) is the intrinsic aqueous solubility of the drug in the absence of excipient, and Saq is the drug solubility at the corresponding excipient level.

### 2.6. Ultra-Performance Liquid Chromatography (UPLC)

Quantification of etoposide in the different samples was carried out using a Waters Acquity UPLC H-Class system (Waters Corp., Milford, MA, USA) equipped with a photodiode array detector and controlled via Empower software, using a method previously described [65]. Chromatographic separation was achieved on an XTerra^®^ C18 column (3.5 μm, 4.6 × 150 mm). Briefly, a gradient mobile phase consisting of water:acetonitrile (both containing 0.1% trifluoroacetic acid) was applied, shifting from 85:15 to 10:90 (*v*/*v*) over a 10 min run time at a flow rate of 0.5 mL/min. The injection volume ranged from 5 to 20 μL. A detection wavelength of 285 nm was used for etoposide quantification.

Excellent linearity (r^2^ = 1) was obtained over a wide (0.1–12 µg/mL) etoposide concentration range. High stability, recovery, accuracy, precision and reproducibility, as well as low etoposide LOD (20 ng/mL) and LOQ (100 ng/mL), were achieved. Both inter- and intraday coefficients of variation were smaller than 1%.

### 2.7. Statistical Analysis

All data are presented as means ± standard deviation (SD). Solubility studies and in vitro PAMPA permeability studies were conducted in quadruplicate (n = 4), whereas SPIP studies in rats were performed in triplicate (n = 3) for each experimental group. Statistical analyses were performed using the nonparametric Kruskal–Wallis test for multiple group comparisons, and the Mann–Whitney U-test for pairwise comparisons, where appropriate. A *p*-value of less than 0.05 was considered statistically significant.

## 3. Results

### 3.1. Solubility

**Figure 1:**
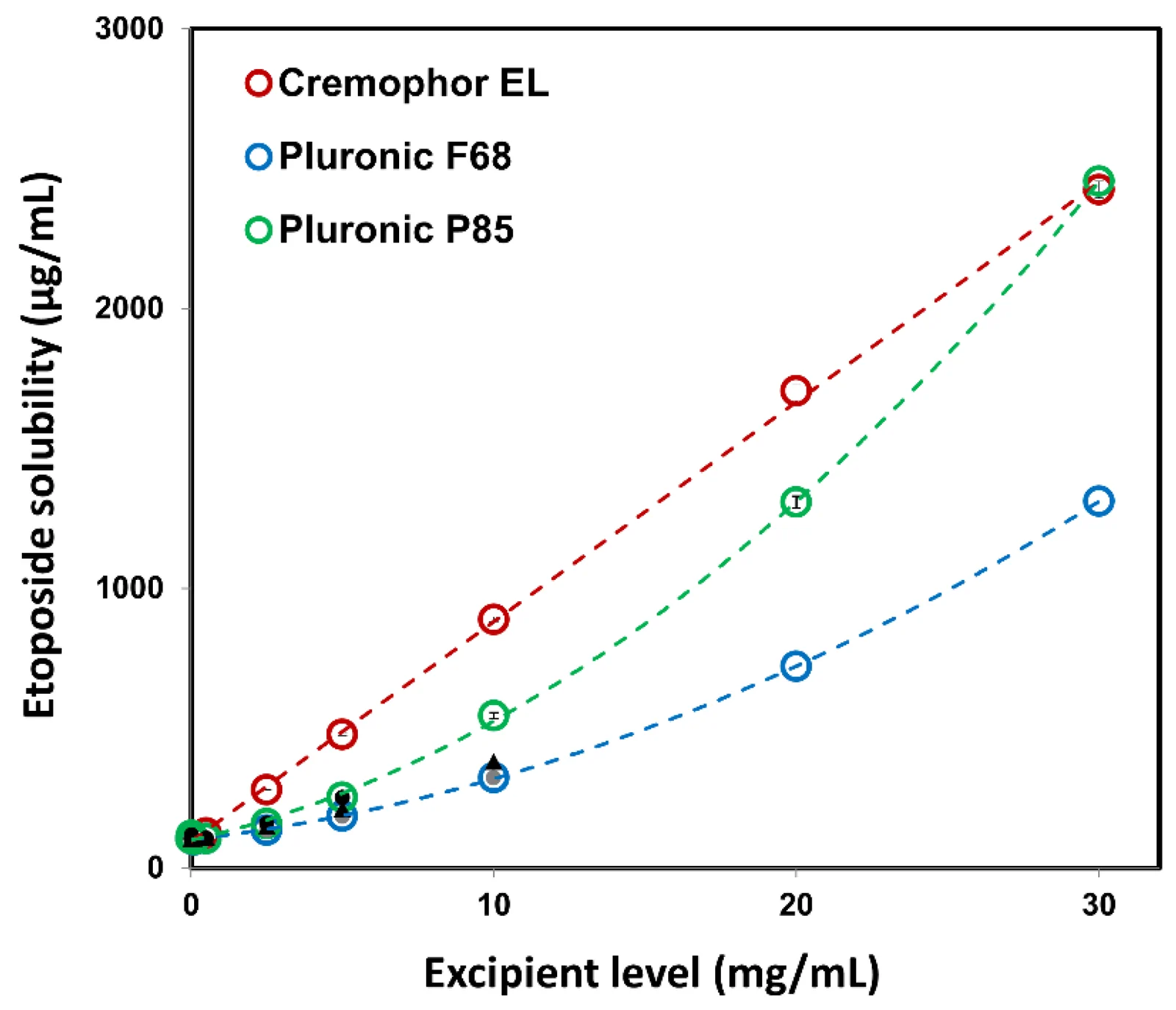
Etoposide aqueous solubility as a function of increasing levels of Cremophor EL (red), Pluronic P-85 (green), or Pluronic F-68 (blue) at room temperature (25 °C). Data presented as mean ± SD; n = 4.

The solubility of etoposide at room temperature (25 °C) in the presence of increasing levels of Cremophor EL, Pluronic P-85, or Pluronic F-68 is presented in Figure 1. It can be seen that all three excipients significantly enhanced the solubility of etoposide in a concentration-dependent manner. A linear drug solubility increase was observed with Cremophor EL, while the solubility increase afforded by both Pluronic excipients was nonlinear. Also, while Cremophor EL and Pluronic P-85 showed comparable solubilization abilities, Pluronic F-68 was less effective in solubilizing etoposide; it can be seen that while etoposide solubility increased from ∼100 µg/mL to ∼2500 µg/mL in the presence of 30 mg/mL of either Cremophor EL or Pluronic P-85, a similar level of Pluronic F-68 allowed half of this drug concentration. The substantial apparent solubility increase demonstrates the reason for the common use of these excipients in solubility-enabling formulations.

### 3.2. In Vitro Permeability Studies Across Artificial Membrane

**Figure 2:**
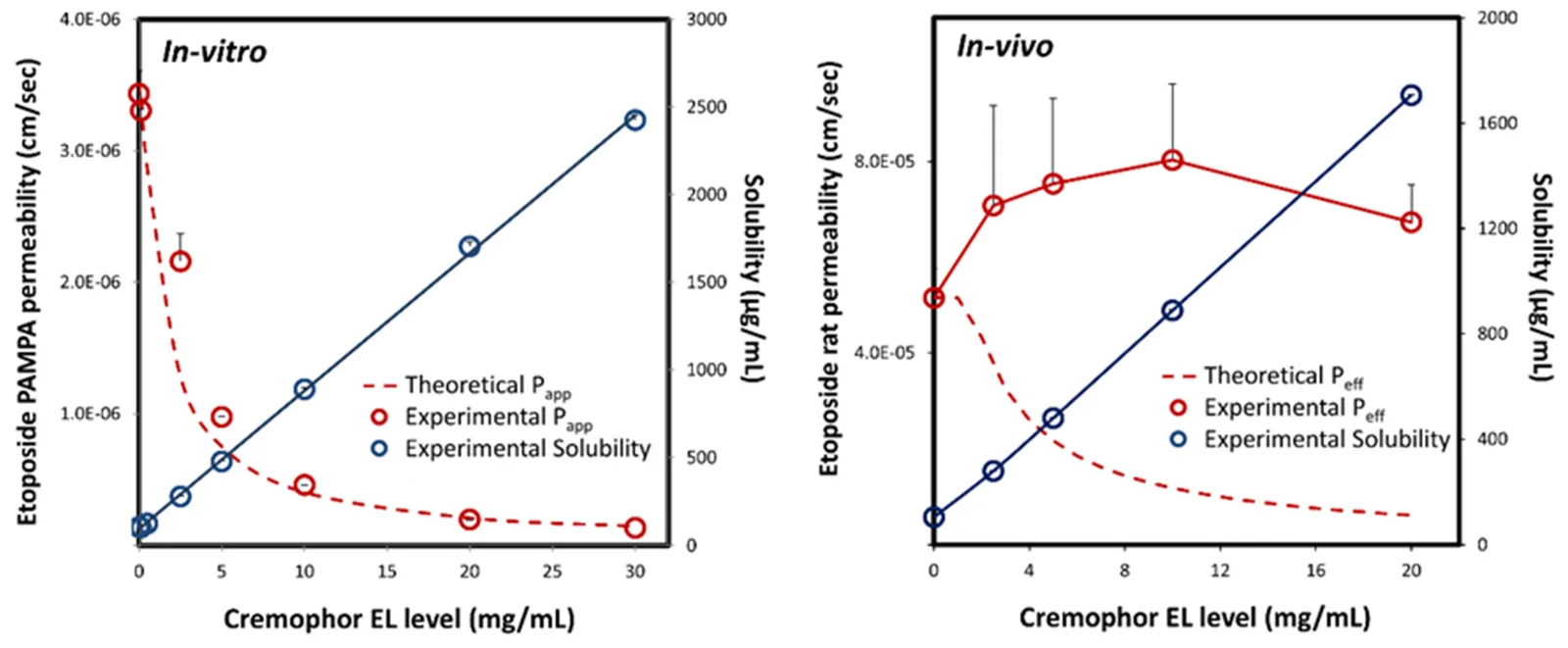
Experimental permeability of etoposide in vitro across artificial membrane (**left panel**) and in vivo across rat jejunum (**right panel**) as a function of increasing levels of Cremophor EL (red circles). Red dashed line, theoretical permeability prediction; blue circles, experimental solubility. Data presented as mean ± SD; n = 3.

**Figure 3:**
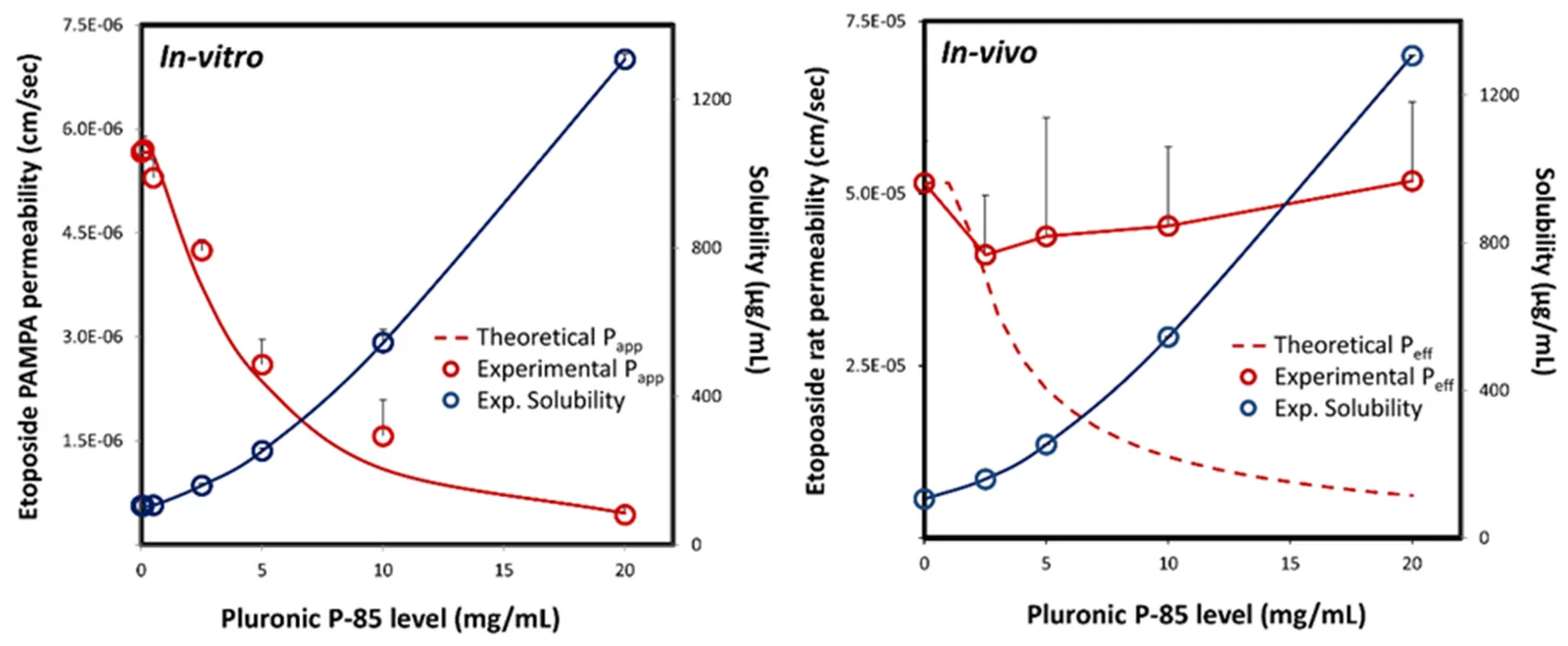
Experimental permeability of etoposide in vitro across artificial membrane (**left panel**) and in vivo across rat jejunum (**right panel**) as a function of increasing levels of Pluronic P-85 (red circles). Red dashed line, theoretical permeability prediction; blue circles, experimental solubility. Data presented as mean ± SD; n = 3.

**Figure 4:**
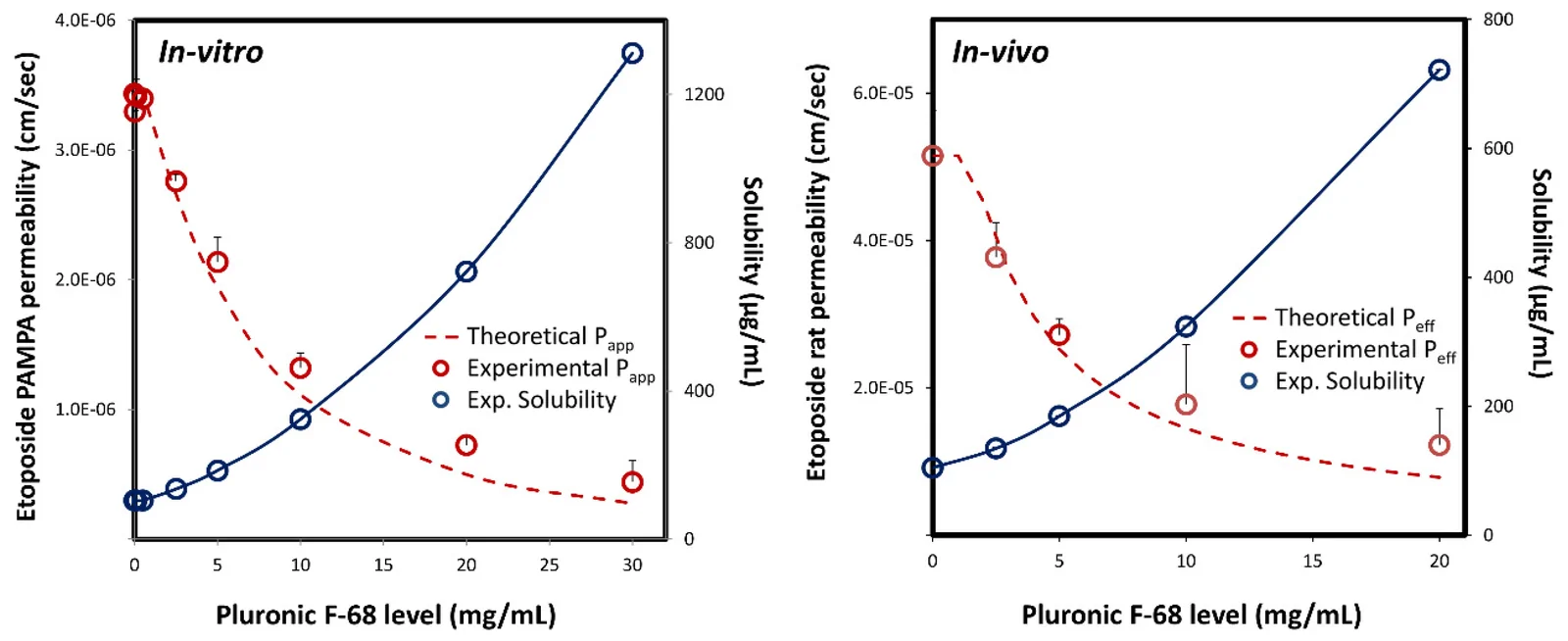
Experimental permeability of etoposide in vitro across artificial membrane (**left panel**) and in vivo across rat jejunum (**right panel**) as a function of increasing levels of Pluronic F-68 (red circles). Red dashed line, theoretical permeability prediction; blue circles, experimental solubility. Data presented as mean ± SD; n = 3.

In vitro PAMPA apparent permeability of etoposide from solutions containing increasing levels of Cremophor EL, Pluronic P-85, or Pluronic F-68 is presented in the left panel of Figure 2, Figure 3 and Figure 4, respectively. A consistent, concentration-dependent decline in PAMPA permeability was revealed with all three excipients; increasing levels of each of the three excipients resulted in decreased Papp. For instance, the intrinsic permeability of etoposide (Papp without excipient) was 3.4 × 10^−6^ cm/s, which dropped 26-fold to 1.3 × 10^−7^ cm/s in the presence of 30 mg/mL Cremophor EL (Figure 2). These results clearly demonstrate the solubility-permeability tradeoff phenomenon: the presence of a solubilizer successfully leads to increased apparent drug solubility, which is accompanied by decreased apparent permeability. Moreover, theoretical concentration-dependent etoposide Papp values for each excipient were calculated using the relationship Pm = Pm(0) × Saq(0)/Saq (signified by the red line on the left panel of Figure 2, Figure 3 and Figure 4), showing excellent correlation with the experimental PAMPA Papp values (red circles on the same figures), for all three excipients. Of course, artificial membranes lack any biochemical components, e.g., transporters; hence, this set of data was complemented by the following in vivo permeability studies in rats.

### 3.3. In Vivo Permeability Studies in Rat

**Figure 5:**
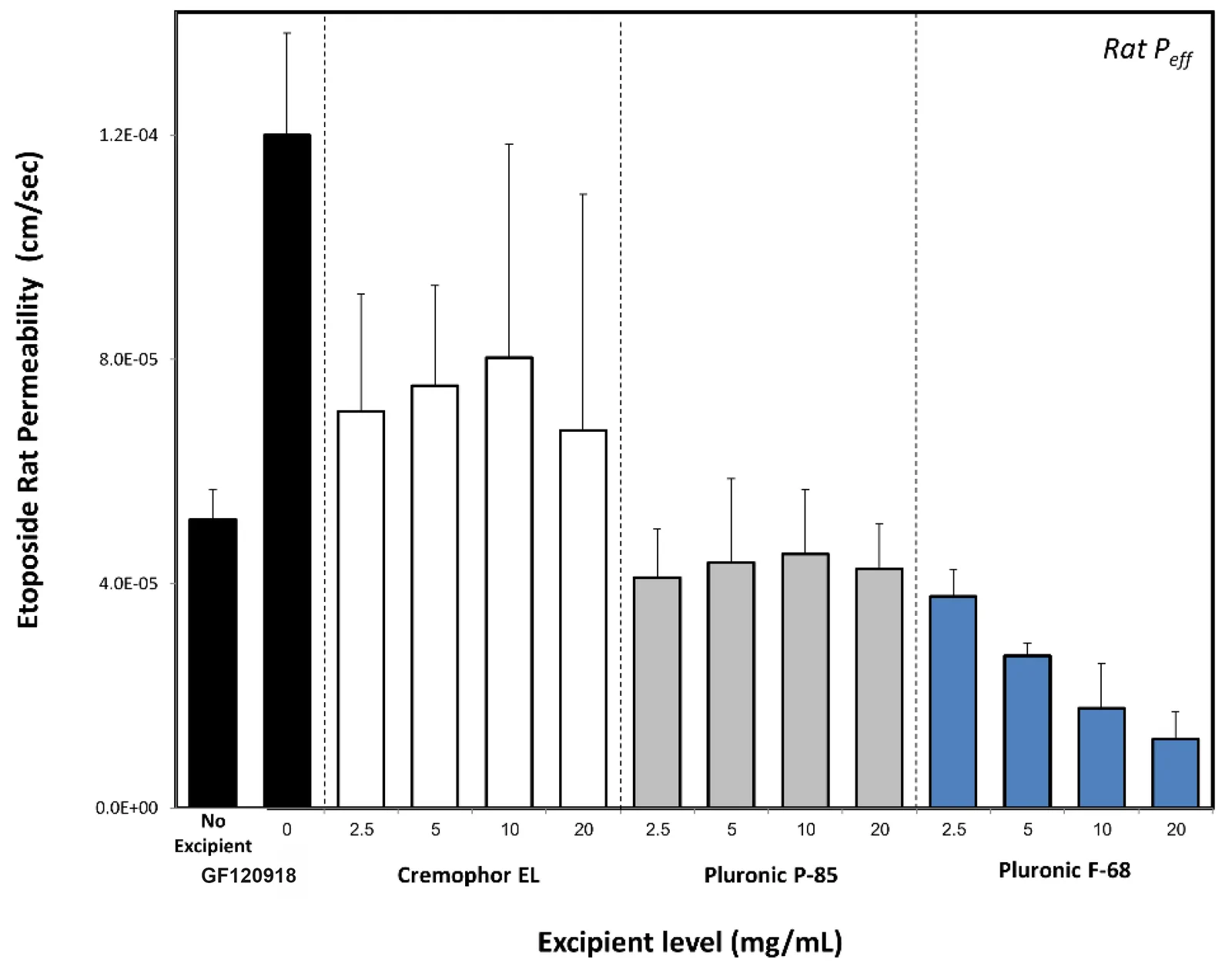
Etoposide rat jejunum permeability without any excipient or in the presence of 20 µM GF120918 (black bars), increasing levels of Cremophor EL (white bars), Pluronic P-85 (gray bars), or Pluronic F-68 (blue bars). Data presented as mean ± SD; n = 3.

Jejunum intestinal permeability of etoposide in rats from solutions containing increasing levels of Cremophor EL, Pluronic P-85, or Pluronic F-68 is presented in the right panel of Figure 2, Figure 3 and Figure 4, respectively. Data are presented as mean ± SD (n = 3 rats per experimental group). Similar to the PAMPA results, increasing levels of Pluronic F-68 (Figure 4) resulted in decreased drug permeability and the typical solubility-permeability tradeoff; it can be seen that etoposide intrinsic permeability of 5.2 × 10^−5^ cm/s dropped 5-fold to ~1 × 10^−5^ cm/s in the presence of 20 mg/mL Pluronic F-68, with good correlation between the predicted (signified by the red dashed line in the right panel of Figure 4) and the experimental Peff values (the red circles on the same figure). Consequently, the excellent match between the in vitro (right panel) and the in vivo (left panel) of Figure 5 is evident. In contrast, the other two excipients resulted in very different results; it can be seen that increasing levels of both Cremophor EL and Pluronic P-85 (right panel of Figure 2 and Figure 3, respectively) were not associated with decreased apparent permeability of etoposide. In contrast to the in vitro Papp results, the intrinsic permeability of etoposide (5.2 × 10^−5^ cm/s) increased ~1.5-fold in the presence of 2.5–10 mg/mL Cremophor EL (Figure 2), highlighting a concomitant gain of both solubility and permeability of etoposide with this excipient. The solubility-permeability tradeoff was also avoided with Pluronic P-85, as etoposide permeability remained unchanged with increasing levels (5–20 mg/mL) of this excipient (Figure 3). The lack of correlation between the predicted permeability (dashed red line) and the experimental Peff values (red circles) with Cremophor EL and Pluronic P-85, as well as between the in vitro (left panel) and in vivo (right panel) results, can be readily appreciated in Figure 2 and Figure 3, respectively. The overall obtained in vivo permeability results with the three investigated excipients are summarized in Figure 5, next to two control groups: etoposide intrinsic permeability (in the absence of any excipient), and the drug permeability in the presence of the strong P-gp inhibitor GF120918 (20 µM). Three different permeability patterns can be readily appreciated in this figure: while increased Peff was obtained with Cremophor EL, unchanged Peff was obtained with Pluronic P-85, and decreased Peff with Pluronic F-68. These different types of solubility-permeability interplay are correlated to the efflux inhibitory intensity exerted by the three excipients.

## 4. Discussion

This study demonstrates that closely related nonionic surfactants, while all capable of significantly increasing etoposide apparent solubility, exhibit markedly different effects on in vivo drug permeability. Pluronic F-68 adhered to the classical solubility-permeability tradeoff, whereas Pluronic P-85 and Cremophor EL overcame this limitation. Specifically, Pluronic P-85 maintained permeability despite increasing solubility, while Cremophor EL produced the most favorable solubility-permeability profile, simultaneously enhancing both parameters. These findings highlight that excipient selection can dictate the success or failure of oral formulations in maximizing etoposide exposure, with Cremophor EL emerging as the most promising candidate among the surfactants tested. More broadly, these results emphasize the importance of considering both solubility and permeability in formulation design, rather than focusing solely on solubility enhancement.

Interestingly, in vivo permeability results for Cremophor EL and Pluronic P-85 did not correlate with in vitro measurements across artificial membranes (Figure 2 and Figure 3). While in vitro PAMPA permeability followed the “classic” solubility-permeability tradeoff, showing decreased etoposide permeability with increasing excipient level, unchanged/increased apparent permeability of the drug was evident in vivo with Cremophor EL and Pluronic P-85. This lack of in vitro/in vivo correlation likely indicates that transporter-mediated processes, absent in artificial membranes, are responsible for the unique individual solubility-permeability interplay of each excipient. Like many other anticancer agents, etoposide is a substrate of efflux transporters; studies have shown that etoposide is a substrate of multiple efflux transporters, including P-gp, MRP2, and BCRP, which are expressed in the intestinal epithelium [38,66,67,68].

Sex-related differences may also influence intestinal permeability and drug absorption in rat models. Previous studies have demonstrated that intestinal permeability and the activity or expression of drug transporters can differ between male and female rats, although these effects appear to be compound- and strain-dependent [69]. In addition, significant sex-related differences in the gastrointestinal environment of male and female Wistar rats were demonstrated including differences in pH, osmolality, surface tension, and P-glycoprotein expression. These physiological differences, particularly the higher intestinal P-gp expression observed in females, may influence oral drug absorption and should therefore be considered in preclinical drug development [70]. Thus, the use of male rats represents a potential limitation of the study.

The enhanced in vivo permeability observed with Cremophor EL and Pluronic P-85 may therefore be attributed to P-gp inhibition, which increases the active transport component (P_Total_ = P_Passive_ + P_Active_), offsetting the potential reduction in passive permeability caused by micellar solubilization.

In contrast to the favorable solubility-permeability interplay observed with Cremophor EL and Pluronic P-85, Pluronic F-68 represents a classical example of the solubility-permeability tradeoff. Pluronic F-68, a hydrophilic and high-molecular-weight surfactant, lacks any P-gp inhibitory properties and, therefore, does not enhance active permeability. The excellent correlation observed between in vitro and in vivo permeability values for etoposide in the Pluronic F-68 formulations (Figure 4) confirms that permeability in this case is governed entirely by passive diffusion, with no contribution from transporter modulation. The observed decrease in etoposide permeability from Pluronic F-68 formulations can be attributed to reduced free drug concentrations available for absorption. Above its critical micelle concentration (CMC), the drug becomes solubilized within micelles, reducing the free fraction of etoposide available for absorption and decreasing passive permeability. This solubility-permeability tradeoff phenomenon observed with surfactants and other types of solubility-enabling formulations can also be explained via the equation P = DK/h, where P is the intestinal permeability, D is the drug’s diffusion coefficient through the membrane, K is the membrane/water partition coefficient, and h is the membrane thickness. Increased aqueous solubility (via micellization) decreases the partition coefficient (K), leading to lower passive permeability.

The favorable solubility-permeability profiles achieved with Cremophor EL and Pluronic P-85 were further demonstrated by comparison with the selective P-gp inhibitor GF120918, which produced the highest in vivo etoposide permeability (1.2 × 10^−4^ cm/s) solely through active transport inhibition. Cremophor EL and Pluronic P-85 yielded the next highest permeability values, whereas Pluronic F-68 produced the lowest, consistent with their transporter-modulating properties. These results support previous findings that nonionic surfactants inhibit P-gp via ATPase modulation [71] rather than complete transporter blockade, in contrast to potent inhibitors like GF120918 [72]. However, because P-gp activity was not directly measured in the present study, these findings should be considered supportive rather than direct evidence of P-gp inhibition.

The P-gp inhibitory potency of the tested surfactants appears to correlate with both hydrophobicity and molecular weight [73]:

- Cremophor EL: HLB 12–14, MW ~2500 g/mol;
- Pluronic P-85: HLB 16, MW ~4600 g/mol;
- Pluronic F-68: HLB 29, MW ~8400 g/mol.

Amphiphilic surfactants such as Cremophor EL and Pluronic P-85 can integrate into the phospholipid bilayer, disrupt membrane integrity, and inhibit P-gp ATPase activity. In contrast, highly hydrophilic, high-molecular-weight Pluronic F-68 lacks sufficient membrane interaction, explaining its minimal effect on permeability.

These mechanistic insights are further supported by our previous studies with vitamin E TPGS [38,39], a low molecular weight (~575 g/mol) highly lipophilic (HLB ~13) surfactant, which effectively penetrates intestinal membranes and fully inhibits P-gp activity, similar to GF120918. Based on this analysis, the relevant excipients can be ranked for P-gp inhibitory potency: TPGS > Cremophor EL > Pluronic P-85 > Pluronic F-68.

Several limitations of the present study should be noted. Although the observed permeability patterns and previous reports suggest a potential role of P-gp modulation, P-gp activity or expression was not directly assessed in this study. Caco-2 transport studies, P-gp ATPase assays, and pharmacokinetic/oral bioavailability studies can confirm the proposed mechanism and determine whether the observed permeability effects translate into increased systemic exposure.

The surfactant concentrations used in the present study were above the reported CMC values of Cremophor EL (0.01%) [74], Pluronic P-85 (0.035% *w*/*w*) [75], and Pluronic F-68 (0.4%) [76], indicating that micellization may have occurred. Although micellization can reduce the free fraction available for membrane permeation, free etoposide concentrations were not directly measured in the present study. Thus, differences in micellization and free drug availability may also contribute to the observed permeability differences.

Overall, this study demonstrates that certain solubility-enhancing excipients, such as Cremophor EL and Pluronic P-85, can increase intestinal permeability in addition to enhancing drug solubility, likely through modulation of intestinal efflux transporters [32,77]. These findings highlight the importance of considering both solubility and permeability when selecting excipients for oral formulation development of poorly permeable drugs. Further transporter-specific and pharmacokinetic studies are needed to confirm the underlying mechanisms and determine whether these effects translate into improved oral bioavailability.

## 5. Conclusions

This work demonstrates that the solubility-permeability tradeoff is not observed to the same extent for all solubility-enhancing surfactants. While all tested surfactants increased etoposide solubility, Cremophor EL and Pluronic P-85 maintained or even enhanced intestinal permeability, whereas Pluronic F-68 exhibited the classical tradeoff behavior. These findings highlight the importance of considering both solubility and permeability, as well as the physicochemical properties of excipients, in the development of oral formulations for poorly permeable drugs. The observed permeability effects likely involve interactions with intestinal efflux transporters and micellization; however, these mechanisms were not directly investigated in the present study. Further transporter-specific and pharmacokinetic studies could determine whether these effects translate into improved oral bioavailability.

## Data Availability

All data generated in this research are presented in the paper and/or available on request from the corresponding author.
